# *De novo *sequence assembly and characterization of the floral transcriptome in cross- and self-fertilizing plants

**DOI:** 10.1186/1471-2164-12-298

**Published:** 2011-06-07

**Authors:** Rob W Ness, Mathieu Siol, Spencer CH Barrett

**Affiliations:** 1 Department of Ecology & Evolutionary Biology, University of Toronto, 25 Willcocks Street, Toronto, ON M5S 3B2, Canada; 2 Centre for the Analysis of Genome Evolution & Function, University of Toronto, 25 Willcocks Street, Toronto, ON M5S 3B2, Canada

## Abstract

**Background:**

The shift from cross-fertilization to predominant self-fertilization is among the most common evolutionary transitions in the reproductive biology of flowering plants. Increased inbreeding has important consequences for floral morphology, population genetic structure and genome evolution. The transition to selfing is usually characterized by a marked reduction in flower size and the loss of traits involved in pollinator attraction and the avoidance of self-fertilization. Here, we use short-read sequencing to assemble, *de novo*, the floral transcriptomes of three genotypes of *Eichhornia paniculata*, including an outcrosser and two genotypes from independently derived selfers, and a single genotype of the sister species *E. paradoxa*. By sequencing mRNA from tissues sampled at various stages of flower development, our goal was to sequence and assemble the floral transcriptome and identify differential patterns of gene expression.

**Results:**

Our 24 Mbp assembly resulted in ~27,000 contigs that averaged ~900 bp in length. All four genotypes had highly correlated gene expression, but the three *E. paniculata *genotypes were more correlated with one another than each was to *E. paradoxa*. Our analysis identified 269 genes associated with floral development, 22 of which were differentially expressed in selfing lineages relative to the outcrosser. Many of the differentially expressed genes affect floral traits commonly altered in selfing plants and these represent a set of potential candidate genes for investigating the evolution of the selfing syndrome.

**Conclusions:**

Our study is among the first to demonstrate the use of Illumina short read sequencing for *de novo *transcriptome assembly in non-model species, and the first to implement this technology for comparing floral transcriptomes in outcrossing and selfing plants.

## Background

Among the most prevalent evolutionary transitions in plants is the shift from cross-fertilization to predominant self-fertilization among numerous angiosperm lineages [1]. This change in mating system has important consequences for many aspects of the biology of selfing taxa including population genetic structure, colonizing ability, genome evolution and the morphology of flowers [2-5]. The loss of floral mechanisms that reduce the incidence of self-fertilization results in high rates of autogamous selfing, leading to the evolution of the 'selfing syndrome' [6-8]. Although studies of the causes and consequences of cross- and self-fertilization in flowering plants have a long and venerable history, beginning with Darwin's seminal work [9], relatively little is known about the underlying molecular changes that accompany the transition from outcrossing to selfing [10,11] and genomic analyses of related outcrossing and selfing plants are in their infancy.

Recent technological advances in DNA sequencing technology have removed several limitations associated with gathering large amounts of genomic data from non-model organisms [12], providing opportunities for detailed investigation into the genomics of mating-system variation and evolution. Although assembling large eukaryotic genomes, *de novo*, may not yet be practical (see [13]), sets of expressed genes or transcriptomes present a viable and attractive alternative to population genetic analyses of whole genome sequences. Transcriptomes represent a fraction of the total genome in size, contain fewer repetitive elements, and by selecting specific tissues they can be enriched for genes relevant to the particular aims of the research. In addition, if the RNA sample is not normalized the relative abundance of different reads has been shown to accurately reflect the expression level of transcripts in the tissue (reviewed by [14]). Despite these potential advantages there remain a number of challenges for *de novo *transcriptome assembly, including gene duplication or paralogy, heterozygosity and alternative splicing, each of which require careful consideration.

There are relatively few studies to date involving *de novo *transcriptome assembly in non-model organisms. So far the majority have used the Roche 454 GS platform (currently 200-400 bp/read, 2-4 × 10^8 ^bp/run) which has the advantage of longer reads, but produces a fraction of the total amount of sequence produced per instrument per run compared with the Illumina GAII platform (currently 38-100 bp/read, 10-20 × 10^9 ^bp/run) (but see [15-17]). Therefore, to maximize the coverage for rare transcripts, cDNA samples are typically normalized. As a result, studies using 454 are not able to estimate expression levels of different ESTs, as this requires deep sequencing of non-normalized cDNA. Further, due to lower sequencing depth many transcripts are represented by a single read, and others by very few reads. This can create problems in accurately distinguishing SNPs from errors, and in retrieving orthologous transcripts for sequence comparisons across experiments or species. Therefore, new methods are required to generate and assemble large datasets, many of which currently consist of substantially shorter reads.

Here, we present *de novo *floral transcriptome assemblies using short read sequencing of four individual plants of neotropical *Eichhornia *(Pontederiaceae) species that vary in floral morphology and mating system. The samples include three individuals of *E. paniculata*, including two from independently derived selfing populations and the third an outcrosser. *Eichhornia paniculata *is an annual diploid that has been the subject of detailed studies over the past two decades on the ecology and genetics of mating-system variation (reviewed in [18]). Populations of *E. paniculata *are largely concentrated in northeastern Brazil, with smaller foci in Jamaica and Cuba and isolated localities in Nicaragua and Mexico. Populations in Brazil are largely outcrossing and possess the sexual polymorphism tristyly, which promotes cross-pollination among the three floral morphs (reviewed in [19]). Morphological, genetic and biogeographical evidence indicates that tristyly in *E. paniculata *has broken down on multiple occasions resulting in independently derived selfing populations [18,20,21]. Populations in Jamaica are largely composed of selfing variants of the mid-styled morph (M-morph) in which short-level stamens are elongated to a position adjacent to mid-level stigmas resulting in autonomous self-fertilization. In contrast, plants in Mexico and Nicaragua are selfing variants of the long-styled morph (L-morph) with a different arrangement of their sexual organs (see figure two in [18]). Although both variants possess the selfing syndrome, comparisons of molecular variation at 10 EST-derived nuclear loci indicate a high level of differentiation consistent with their separate origins from different outcrossing ancestors (see figure three in [18]). Our analysis included both of these selfing variants, an individual of an outcrossing L-morph from northeastern Brazil, and a selfing individual of *E. paradoxa*, the sister species of *E. paniculata *[22,23]. We included *E. paradoxa *to serve as an additional selfing phenotype and as an outgroup for future studies of molecular evolution. The approaches we describe demonstrate the utility of short-read sequencing for quantifying variation in gene expression among related samples.

## Methods

### Sampling & RNA preparation

We selected the four plants used in our study from glasshouse collections maintained at the University of Toronto. The plants were originally obtained by germinating open-pollinated seed collected from field populations at the following localities: outcrossing L-morph B211, Fortaleza, Ceará, N.E. Brazil; selfing M-morph J16, Georges Plain, Westmoreland, Jamaica; selfing L-morph N1, Rio Las Lajas, Rivas, Nicaragua; *E. paradoxa*, Patos, Paraíba, N.E. Brazil. We collected fresh tissue from different stages of bud and flower maturation to sample as many of the genes expressed during development as possible. To standardize sampling across all four genotypes flower buds were classified into six sizes (< 3 mm, 3-5 mm, 5-7 mm, 7-10 mm, > 10 mm, open flower), with multiple buds for each stage and each stage represented equally among all individuals. Following bud and flower removal, samples were immediately frozen in liquid nitrogen to avoid RNA degradation. We extracted RNA from pooled bud samples from each individual using the Invitrogen (Carlsbad, CA) Trizol high salt precipitation extraction protocol. We visualized RNA extracts on a gel to provide an initial assessment of quality and quantified using a spectrophotometer.

### Sequencing

We provided 5 μg of total RNA to the Center for Analysis of Genome Evolution & Function (CAGEF) at the University of Toronto (Toronto, ON) for sequencing. The RNA was sequenced using the Illumina (San Diego, CA) mRNA-Seq, paired-end protocol on a Genome Analyzer, GAII, for 40 cycles. This resulted in an average of ~38.9 × 10^6 ^total reads per sample or ~1.55 × 10^9 ^bp of sequence per sample (Table 1). It is common for the quality of bases from the 3' end of Illumina reads to drop in quality, we therefore trimmed the 3' end of reads when the Phred quality score dropped below *Q *= 20 (or 0.01 probability of error) for two consecutive bases. In addition, we also trimmed all 5' and 3' stretches of ambiguous 'N' nucleotides. Trimming resulted in reads with a mean length of 37.4 bp across all samples and a minimum length of 20 bp was applied during sequence trimming.

**Table 1:** Sequencing statistics for this study.

Sample	Number of total reads	Total raw Bases	Reads after trimming	Bases after trimming
*E. paradoxa - *Brazil	39,198,840	1,567,953,600	24,539,860	812,538,461
*E. paniculata *- Brazil	38,374,454	1,534,978,160	24,796,504	827,426,981
*E. paniculata *- Jamaica	39,047,450	1,561,898,000	23,555,014	764,543,073
*E. paniculata *- Nicaragua	38,980,224	1,559,208,960	22,323,972	740,370,472

The number of reads and total amount of sequence generated for each sample of *Eichhornia*. Values are shown for both raw sequence and the sequence after trimming from reads with low quality bases.

### *De Novo* assembly

We performed *de novo *assembly on each sample separately using software packages designed for short read sequence assembly including Abyss [24], Edena [25], Velvet [26] and Oases (D. R. Zerbino, European Bioinformatics Institute). To choose the optimal parameters for each method, we used a combination of BLASTx searches of the NCBI non-redundant protein database (NR), and summary statistics of the assemblies (*N50*, longest contig, number of contigs, proportion of reads assembled). Consideration of the summary statistics led us to finally choose Oases, which generated the longest assembled ESTs, with the best hits to NR in terms of low E-values. Oases is a program designed as an extension of Velvet, specifically released for assembly of transcriptome sequences. Unlike the other software mentioned above, Oases handles the uneven coverage of contigs due to variation in expression levels of the transcripts in the sample. We assembled each sample using the same assembly parameters (K-mer length = 25, coverage cutoff = 10, minimum contig length = 100 bp). A consequence of the algorithm in the the version of Oases we used was a tendency to generate identical or near-identical contigs, possibly due to allelic variants or sequencing errors. To lower redundancy in the dataset we removed these by comparing each transcriptome assembly to itself using BLAST [27,28]. Any pair of contigs that were > 99% identical over 95% of the length of the shorter contig were collapsed into a single contig.

### Consensus transcriptome generation

To create a reference transcriptome we conducted a 'four-way' reciprocal BLAST (all pairwise comparisons) to identify all orthologous sequences. The goal here was to identify sequences that may not show similarity to other known proteins or ESTs, but which are expressed in more than one sample. This procedure allowed us to confirm a large proportion of our transcripts without having to rely on comparative searches to distantly related species. In addition, we were able to generate longer consensus sequences when one of the reciprocal best BLAST sequences was longer than the others. This was implemented using a custom Biopython script [29] and BLAST.

We compared each of the four individual redundancy-reduced transcriptome assemblies to each other using BLASTn (default parameters without low complexity filter). Reciprocal best BLAST hits found in more than two samples were then placed into groups and aligned using Muscle [30] to generate a consensus sequence. We defined a number of criteria to identify orthologous sequences including minimum alignment length (200 bp), minimum sequence identity (90%), and minimum alignment proportion (> 80% of shorter sequence). This last criterion was used to avoid alternatively spliced transcripts or incompletely aligned contigs being collapsed in an alignment. After generating the consensus sequences with reciprocal BLAST we identified unaligned sequences that aligned well to the ortholog groups, but may not have been > 200 bp. These sequences were incorporated into the consensus only when the contig extended the length of the consensus sequence, and had > 95 % identity over > 50 bp with no unalignable segments.

Due to low coverage or repetitive elements within coding loci it is possible that separate contigs are fragments of a single protein. To reduce fragmentation and recover longer coding sequences we aligned each contig to all unique *Oryza sativa *(another monocotyledon) proteins using BLASTx. We used *O. sativa *because it is the closest related plant for which an extensive set of protein sequences is available. This allowed us to identify consensus sequences that probably belong to the same protein and assemble them into a single contig. We aligned sequences that were potentially from the same protein enabling an elongated consensus to be generated. Only a small number of contigs were found to be potentially fragments of longer ESTs (~1.6%) and all of the alignments made in Sequencher 4.7 were verified manually to ensure that no gaps, or mismatches were introduced.

After we assembled the consensus of all potential orthologs we identified sequences that were not included in these groups, but had homologs in other species (hereafter referred to as singletons). We compared each singleton against NR and those over the size threshold of 1000 bp and with a strong BLASTx hit (expectation or *E*-value < 1 × 10 ^-15^) were included in the reference sequence along with all potential orthologs identified with our reciprocal BLAST scheme. To ensure that there was no remaining redundancy of transcripts in the consensus we used the same technique to reduce redundancy, as outlined above. The assembled sequences have been deposited in the NCBI's transcriptome shotgun assembly (TSA) database.

### Genotype calling & SNP detection

For each sample we mapped the original short reads, trimmed using the base qualities as outlined above, to the consensus transcriptome using the Burrows-Wheeler Aligner (BWA) version 0.5.7 [31]. By mapping the reads back to the consensus transcriptome we were able to more accurately estimate coverage and use counts of nucleotides at each position to call genotypes. BWA allowed us to vary the number of mismatches between the reference and aligned reads. We tested a number of parameter values for alignment and all analyses presented here use a value of *n *= 0.05, where *n *is the fraction of missing alignments given 2% uniform base error rate. To generate a final sequence for each individual we included all loci where coverage was on average greater than five fold across the locus. Furthermore, within loci only sites where coverage exceeded five were used to call genotypes, the other sites were marked as ambiguous.

To generate genotypes for each sample we used the genotype calling method implemented in the software Maq [32]. This method takes into account the counts of different bases at each site, as well as the quality of each base and the mapping quality of the sequence read. Maq uses a Bayesian statistical model to compare the inferred genotype to the original reference. To call genotypes we used a threshold 'consensus quality' cutoff of *Q *> 13 (*P *= 0.05), where *Q *is the Phred-scaled probability that the consensus genotype call is wrong [32-34]. Sites for which we could not determine the consensus, with at least this level of confidence, were marked as ambiguous. To detect potential errors in read mapping we assumed that selfing genotypes were largely homozygous, and therefore the presence of heterozygous sites in multiple selfing genotypes may indicate errors in read mapping (see Discussion). These loci were excluded from downstream analyses. True heterozygosity was therefore estimated at loci where there was no evidence of read mapping errors. We used a subset of the identified loci that were shared between all four samples to assess the number of SNPs between pairs of genotypes. We also calculated nucleotide polymorphism values, *θ_W _*[35] for the three *E. paniculata *sequences

### Measurement of gene expression

In addition to generating a consensus sequence, we also used abundance of reads derived from each locus to estimate gene expression. We calculated the number of fragments per kilobase per million fragments mapped (FPKM) with the program Cuffdiff from the package Cufflinks v 0.83 [36]. This program estimates confidence intervals around expression estimates of each transcript using a Bayesian inference method and will identify significant differences in expression using a FDR control and Benjamini-Hochberg correction for multiple tests [37]. To compare expression differences among selfing genotypes, we identified loci in which both *E. paniculata *selfing genotypes, or all three selfing genotypes (including *E. paradoxa*), differed in expression relative to the outcrossing genotype of *E. paniculata *from Brazil. To estimate the overall similarity in expression we calculated the correlation of FPKM among pairs of plants after log transformation so that the data fitted a normal distribution. To test for significant differences among correlation coefficients we bootstrapped the data (10,000 replicates) to estimate 95% confidence intervals.

### Functional annotation

To functionally annotate each gene and to assess the quality of our assembly we used the Gene Ontology (GO) based annotation suite BLAST2GO v2.4.2 [38,39]. BLAST2GO allowed us to identify similarity of the sequences in our reference transcriptome to known and predicted proteins, and to assign each of the sequences GO terms that were associated with the proteins found by BLASTx. We searched all 26,994 sequences against the non-redundant protein database (NR) with maximum *E*-value = 1 × 10^-15^. BLAST2GO assigned GO terms using a pro-Similarity-Hit-Filter of 15, an annotation cut-off of 55 and a GO weight of 5. We conducted enrichment analyses to test for an excess or paucity of gene classes (based on GO terms) in test sets relative to the whole reference transcriptome. These included: 1) genes absent in the two selfing *E. paniculata *genotypes (Jamaica and Nicaragua); 2) genes absent in the outcrossing genotype (Brazil), and 3) genes with low expression in the two selfing *E. paniculata *genotypes, and 4) genes with high expression in the two selfing *E. paniculata *genotypes. Additionally, we repeated these contrasts with *E. paradoxa *included in all sets with the Jamaican and Nicaraguan selfers. However, the results of these analyses were not informative because there were no over-represented gene classes in each group, and the results are therefore not presented here. All comparisons were implemented in BLAST2GO, which uses a Fisher's exact test to determine significance after controlling for multiple tests with a false discovery rate (FDR) = 0.05.

### Assessment of the accuracy of EST assembly

To assess the quality of our assembly, we compared the ESTs assembled in our consensus transcriptome with a set of 217 unique ESTs sequenced from *E. paniculata *with Sanger sequencing. These 'Sanger-ESTs' were sampled from a cDNA library generated from leaf and floral tissue (details in [40]). We assembled and aligned forward and reverse strands of each EST using Sequencher 4.7 and edited chromatographs and alignments manually. We compared the two sets of ESTs to identify conflicts, in which pairs from each EST set share a significant portion of their sequence but cannot be aligned over another overlapping portion. This could be an indication of our Illumina ESTs having been assembled incorrectly, creating chimeric sequences of distinct transcripts. Because the set of Sanger ESTs was not exhaustive, we attempted to identify well-assembled ESTs from the subset with conflicting alignments by comparing them to the NR protein database to determine whether there is a known protein that covered the full length of the Illumina EST.

## Results

### Assembly & consensus transcriptome generation

**Figure 1:**
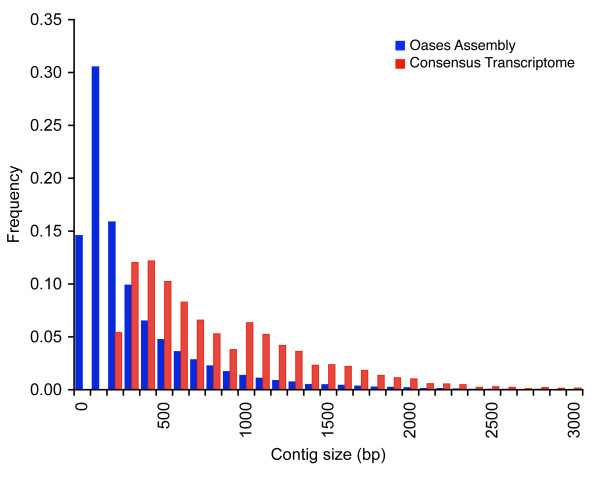
**Histogram of the frequency of different contigs sizes in transcriptome assemblies of *Eichhornia *samples**. Blue bars represent the distribution of contig sizes for the initial *de novo *Oases assemblies and the red bars represent the final consensus transcriptome after being processed in our pipeline. The distribution has shifted to the right indicating more long contigs.

*De novo *assemblies of each sample using Oases resulted in an average of 56,791 (50,581 - 61,922) contigs per sample, totaling approximately 21.3 Mbp of sequence for each individual. Many of the sequences were small, resulting in an *N50 *size of 611 bp and mean contig size of 374 bp (Figure 1). After removing very similar sequences and contigs that were shorter than 100 bp, the mean number of contigs per sample was 44,614, totaling on average 17.6 Mbp/sample.

The four-way reciprocal BLAST scheme returned consensus sequences of all contigs present in at least two samples. This resulted in a nearly two-fold reduction in the total number of contigs to 22,630, along with an increase in the *N50 *to 807 bp and only a slight decrease in the total amount of sequence in the transcriptome, relative to each individual Oases assembly (15.9 Mbp). We attempted to improve our consensus transcriptome by incorporating the remaining contigs into the consensus and by joining contigs that were fragments of the same protein. These steps had only a minor effect on our consensus, increasing the *N50 *to 819 bp, the total length to 16.0 Mbp and decreasing the total number of contigs to 22,282.

BLAST identified all the unincorporated contigs that had similarity to known proteins. 5624 contigs over 1000 bp had BLASTx hits with *E *< 1 × 10^-15 ^to known proteins and were added to the consensus transcriptome. After we removed all remaining redundancy, the final consensus transcriptome consisted of 26,994 contigs, representing 23.9 Mbp of sequence with an *N50 *of 1129 bp and a mean contig size of 884 bp. The final consensus transcriptome resulted in a nearly two-fold increase in the *N50*, was comprised of ~30,000 fewer contigs than any of the original assemblies, and represented a slightly larger total transcriptome length (Table 2).

**Table 2:** Summary statistics for reference transcriptome through progressive stages of assembly.

Assembly stage	*N50*	Mean contig size	Total length	Number of contigs
Oases assembly	611	374.1	21,311,238	56,971
Reciprocal BLAST	807	703.5	15,919,812	22,630
Contig elongation	812	709.9	16,065,194	22,630
Reduced fragmentation	819	717.8	15,995,086	22,282
Final (including singletons)^1^	1,129	884.3	23,869,762	26,994

^1 ^Singletons over 1000 bp with BLASTx hits where *e *< 1 × 10^-15 ^are included in the final consensus

For the first stage all statistics are the average across the four independently sequenced genotypes.

### Genotype calling & SNP detection

Using our consensus transcriptome as a reference, we mapped the original short sequence reads for each sample with the software bwa-0.5.7. On average there was 22.0 reads covering each position in the reference with a standard deviation of 32.0 (Table 3). We generated, on average, 23,543 contigs for each sample. 18,063 of the 26,994 original contigs were found in all four samples and only 139 of the reference contigs were not recovered in any of the samples, likely due to low coverage. Few loci were unique to any single *E. paniculata *genotype, 5254 sequences were shared by all *E. paniculata *samples and not found in *E. paradoxa*. Moreover, 1392 loci were unique to *E. paradoxa*.

**Table 3:** Summary statisitcs for read mapping.

Sample	Number of loci	Mean coverage (per bp ± S.D.)	Number of heterozygous loci	Total sequence (bp)
*E. paradoxa *- Brazil	20,653	21.8 ± 33.7	3,994	17,308,982
*E. paniculata *- Brazil	24,849	24.3 ± 34.1	4,979	21,362,493
*E. paniculata *- Jamaica	24,527	21.7 ± 32.1	1,659	20,943,070
*E. paniculata *- Nicaragua	24,142	20.5 ± 27.9	895	20,603,010

Results of read mapping using the original trimmed short reads for each of the four *Eichhornia *samples against the reference transcriptome

We identified heterozygous loci and potential read mapping errors as loci with one or more bases called as heterozygotes. Assuming that selfing genotypes are largely homozygous, the presence of heterozygous sites in multiple samples of selfers may indicate errors in read mapping (see Discussion). We identified 15,962 loci where there was no evidence of read mapping errors, 8469 loci with some evidence for read mapping errors, and 2563 loci were expressed in either one or zero selfers, precluding the application of this test. For the loci in which there was no evidence for read mapping errors, the number of heterozygous loci was highest in the outcrossing *E. paniculata *genotype (4979) compared to the two selfing genotypes from Jamaica (1659) and Nicaragua (895). *Eichhornia paradoxa *had an intermediate number (3994) of heterozygous loci. To detect the number of SNPs between pairs of genotypes, we selected a conservative set of 5,011 loci (4.2 Mbp) that were expressed in all four individuals and were homozygous in all selfers (Table 4). The outcrossing Brazilian and selfing Nicaraguan genotypes had the fewest divergent sites (36,998). Intraspecific variation in *E. paniculata *was substantially lower than the divergence of each *E. paniculata *sample to *E. paradoxa*.

**Table 4:** Number of single nucleotide polymorphisms (SNPs) between pairs of *Eichhornia *genotypes.

Sample	*E. paradoxa*	*E. paniculata*		
		
		Brazil	Jamaica	Nicaragua
*E. paradoxa - Brazil*	-	0.048	0.048	0.047
*E. paniculata *- Brazil	202,687	-	0.014	0.009
*E. paniculata *- Jamaica	200,576	58,410	-	0.012
*E. paniculata *- Nicaragua	195,967	36,998	51,409	-

Values reflect the polymorphism statistics for 5011 loci that were present in all four samples and homozygous in the three selfing genotypes. Values below the diagonal show the total number of SNPs and values above the diagonal show the number SNPs per bp (4,207,280 bp total).

### Functional annotation

**Figure 2:**
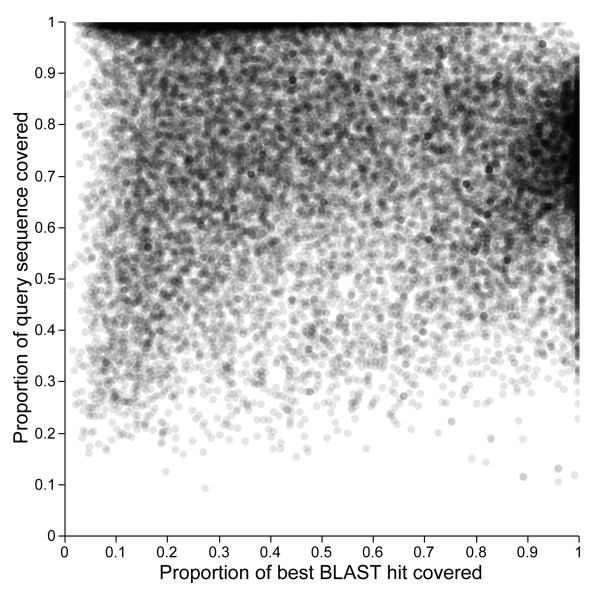
**Proportion of each of the assembled sequences that is aligned to its top BLASTx protein hit versus the proportion of the top hit which is covered by the assembled query sequence**. This plot demonstrates the size of our assembled transcripts relative to their homologs in other plant species. Dense clusters of points along the top of the figure represent loci entirely aligned to their respective protein hits and points along the right are genes fully covering their best BLAST protein hit.

**Figure 3:**
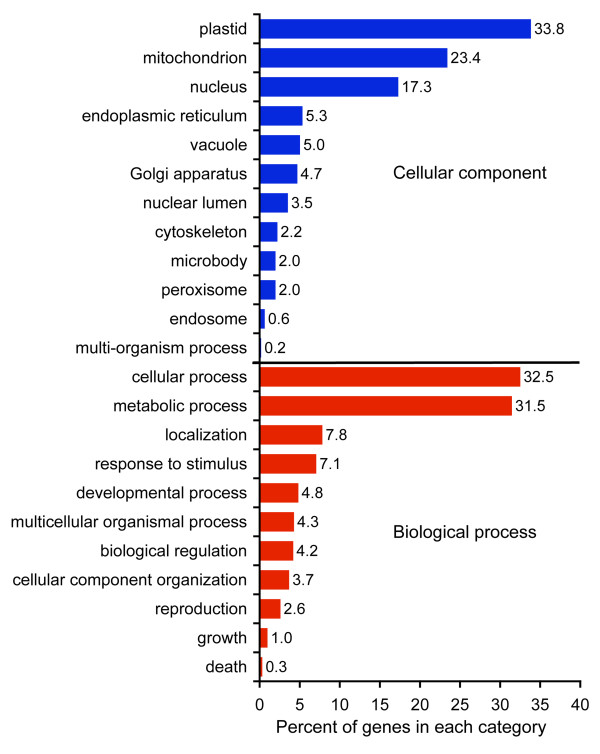
**Distribution of genes in the transcriptome assembly assigned to broad GO categories**. Cellular components (blue) and biological processes (red). Percentages indicate the proportion of sequences assigned within each subcategory using the program BLAST2GO, which assigns putative function to each transcript based on similarity to proteins with GO annotations from other organisms.

23,476 of 26,994 contigs (86.97%) had similarity to known proteins in the NCBI NR database, with a cutoff of *E *< 1 × 10^-10^. 6329 loci had alignments which covered more than 80% of the top protein hits and 10,323 of the query sequences were at least 80% covered by their best protein hit (Figure 2). BLAST2GO assigned a functional annotation to 21,779 of the loci (80.68%). Within the broad GO category 'cellular components', over a third of the sequences were localized to the plastid, 23.4% to the mitochondrion and 17% to the nucleus (Figure 3). A number of other cellular components made up the remaining 25.4 % of the annotated loci. Within the broad GO category 'biological process' the two most common type of genes were those involved in cellular (32.5%) and metabolic (31.4%) processes (Figure 3). 812 genes that were identified are involved in reproductive processes including flower development (269 genes) and pollination (60 genes).

### Gene expression

**Figure 4:**
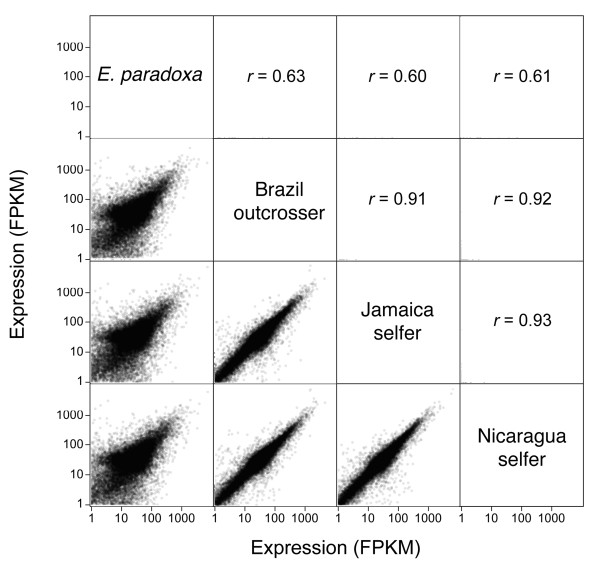
**Pairwise correlations of gene expression between the four genotypes: *Eichhornia paniculata *- Brazil, Jamaica and Nicaragua and *Eichhornia paradoxa***. Above the diagonal are the correlation coefficients for data plotted below the diagonal. Each pair below the diagonal is expression level plotted on a log scale, measured in FPKM, which estimates gene expression from the number of reads that are derived from each transcript in our samples. All correlations are significant at *P *< 0.00001.

Using FPKM to measure gene expression, we found significant correlation in expression among our samples (Figure 4). As expected, the correlation of each of the three *E. paniculata *samples with *E. paradoxa *was lower (*r *from 0.60 - 0.63) compared with the correlation of *E. paniculata *genotypes with one another. The two independently derived selfing genotypes were slightly more correlated (*r_JAM-NIC _*= 0.93), but not significantly more so than either was to the outcrossing genotype from Brazil (*r_BRA-JAM _*= 0.91, *r_BRA-NIC _*= 0.92). There were 147 genes that were significantly up-regulated in all three selfing genotypes compared with the outcrosser, 12 of which were involved in reproduction. A similar number of genes (134) were down-regulated in the selfers relative to the outcrosser, 10 of these genes were involved in pollination or flower development (Table 5).

**Table 5:** Pollen and flower development genes that were differentially expressed in both independently derived selfing genotypes of *Eichhornia paniculata *and were also identified from selfing *Eichhornia paradoxa*.

Homolog name	Fold expression change^1^
^2^Gibberellin receptor (*GID1c*)	0.51****
^2^Peptide transport protein (*PTR1*)	0.62****
^2^*ERECTA*-like 1 (*ERL1*)	0.64*
^2^*DICER*-like1 dsrna-specific nuclease (*DCL1*)	0.77**
^2^Exocyst complex component (*SEC5*)	1.29*
^2^SHK1 binding protein 1 (*SKB1*)	1.38**
^2^*REBELOTE *(*RBL*)	1.64**
^2^Cellulose synthase (*CSLD3*)	0.55***
Pollen-pistil incompatibility 2 (*POP2*)	0.62****
*ECERIFERUM *1 (*CER1*)	0.70***
Auxin signaling F-box 2 (*AFB2*)	0.71****
Transport inhibitor response 1 (*TIR1*)	0.89*
Beta-amylase (*BAM1*)	0.99****
Auxin response factor 8 (*ARF8*)	1.08****
*PEPPER *nucleic acid binding protein (*PEP*)	1.15**
Suppressor of *FRIGIDA4 *(*SUF4*)	1.18*
Phosphoglucose isomerase (*PGI1*)	1.35**
Auxin response factor 6 (*ARF6*)	1.50**
Regulatory particle non-atpase 10 (*RPN10*)	1.50*
Phosphatidylglycerolphosphate synthase 1 (*PGP1*)	1.60*
myb transcription factor (*myb24*)	1.79****
Glutaminyl-tRNA synthetase (*OVA9*)	1.93****

* *p *< 0.05, ** *p *< 0.01, ***p < 0.001, ****p < 0.0001

^1 ^Measured as the mean ratio of expression (FPKM) in each selfer relative to the outcrossing genotype

^2 ^These genes were differentially expressed in the selfing genotypes of *E. paniculata *only

### Gene ontology enrichment tests

We investigated whether there was an excess or paucity of particular gene classes that were differentially expressed in the two selfing genotypes of *E. paniculata *compared to the outcrosser. In the up-regulated genes of the *E. paniculata *selfers there were 146 GO categories that were significantly enriched. However, using a two-tailed Fisher's exact test we found that only genes involved in photosynthesis (photosynthesis GO:0015979, thylakoid GO:0009579, plastid GO:0009536) were significantly over-represented and the remaining 145 classes of genes were under-represented. In the genes that were expressed at a lower level or were completely absent in the selfers, 12 of 106 and 8 of 62 GO classes were significantly over-represented. Significantly, of the 12 classes of genes in the two *E. paniculata *selfers that were over-represented among the down-regulated genes, five are related to the regulation of cellular structure and development (regulation of cell morphogenesis GO:0022604, regulation of cellular component organization GO:0051128, regulation of developmental process GO:0050793, regulation of anatomical structure morphogenesis GO:0022603, regulation of cell size GO:0008361). In the *E. paniculata *selfers, the genes involved in flower development were significantly under represented in both higher and lower expression genes.

### Assessment of EST assembly accuracy

Comparisons of our consensus transcriptome ESTs to a set of 217 Sanger sequenced ESTs revealed 11 Illumina ESTs (5.1%) that conflicted with their best Sanger EST match. These 11 ESTs each had regions that could not be aligned with the full length of the best BLAST hit from the Sanger ESTs and also did not have full-length BLAST hits to known proteins in NR. However, all 11 had highly significant hits to proteins in NR. None of the 11 ESTs appeared to be chimeric assemblies of distinct proteins that may have resulted from errors during assembly. Moreover, 9 of the 11 ESTs had sequence flanking their protein-coding region with significant similarity to known 5' and 3' untranslated regions from other monocot genomes.

## Discussion

We assembled ~24 Mbp of transcriptome sequence in each of four individuals of two species of *Eichhornia*. The data represent an important genetic resource of nearly 27,000 transcripts, many of which are common to all four samples. Further, using read mapping to a consensus transcriptome we have generated statistically informed genotypes for each individual in our study. By choosing to extract RNA from buds and mature flowers we were able to recover many genes involved in reproduction and floral development, some of which are likely to provide future insights on genetic changes to floral traits governing mating-system variation. We now compare our assembly and analysis with previous *de novo *transcriptome sequencing projects and briefly review some of the challenges and interpretations specific to our assembly. In addition, we also discuss the utility of short-read sequencing for characterizing genetic changes in transcriptomes and the expression level of different loci.

### Assembly & consensus transcriptome

Our study is among the first published attempts at a *de novo *transcriptome assembly using short-read (Illumina GAII) sequencing. Although there are now many studies using next generation transcriptome sequencing, most have used the Roche 454 platform (but see [15-17]). As previously mentioned, this platform has the advantage of longer reads but at the expense of less sequence data per run. Longer reads may be critical for resolving assembly challenges associated with repetitive elements, such as gene duplications, allelic differences and alternative splicing (reviewed in [41]). However, despite using shorter reads, our assembly is comparable to other published transcriptomes, which at this time average 37,286 contigs (12,883 - 72,977, *n *= 19 studies), a number similar to our own (26,994 contigs). In addition, despite the longer read lengths (~ 400 bp; 197 - 581 bp, *n *= 19 studies), of previously published transcriptome studies using the Roche 454 platform, our assembly generated contigs that were over twice as long averaging 884 bp. One of the reasons that this is the case is because the Illumina generates greater depth of sequencing thus ensuring more complete coverage of the transcriptome.

Although summaries of the distribution of contig lengths are informative, the ultimate goal of transcriptome assembly is not long sequences, but accurate assembly of full-length sequences. However, it is difficult to assess the success of an assembly without *a priori *knowledge of the transcriptome. One metric that may be informative is the proportion of contigs that have significant similarity to known proteins. It is difficult to compare this measure across studies because each reports slightly different results using different BLAST parameters and databases. However, nearly 87% of our contigs had matches in NR and this value is as high or higher than all other comparable statistics reported in other *de novo *assemblies. Another useful metric is the proportion of the contig and its corresponding BLAST hit that align to one another (Figure 2). 7273 (26.9%) contigs cover greater than 75% of their best BLAST hit and 12,659 (47%) contigs are fully covered by their best BLAST hit. This means that although we assembled a large number of full-length proteins, many of the contigs appear to be fragments of larger proteins. One explanation is that gene duplication or alternative splicing creates repetitive elements in the assembly and these cannot be resolved. Although we found a fraction of our ESTs (5.1%) that had conflicts with Sanger sequenced ESTs, this is likely an overestimate of assembly error because some of these conflicts could result from paralogy or alternative splicing. There was no evidence that any of the 11 conflicting ESTs were chimeric assemblies of two or more proteins and most of the conflicts (9 of 11) appear to be the result of misaligned untranslated regions flanking coding regions. It is possible that some of the discrepancies between the ESTs we assembled and Sanger ESTs or known proteins are true differences, for example, paralogous transcripts or alternatively spliced isoforms.

### Genotype calling & SNP detection

One of the major challenges in dealing with very short sequence reads is that they must be assembled into longer contigs based on overlap with other reads. The algorithms used in many *de novo *assemblers, including Velvet [26], may misinterpret small differences between alleles (SNPs) or gene duplicates as sequencing errors. If this occurs they can be 'collapsed' or purged from the assembly. Although the program Oases has begun to address this problem for transcriptome data, we chose to use read mapping to a consensus transcriptome because it allowed us to use allele frequencies at each site to statistically determine the genotype of each individual [32]. From this approach we obtained, on average, more than 20 reads for each position to inform genotype calls. This allowed us to generate sequences for ~23,500 loci/genotype, 18, 000 of which were found in all samples. An additional benefit of using this approach is that we were able to identify heterozygous loci and potential read mapping errors. Of the 26,994 consensus sequences, 8712 heterozygous loci were identified. As expected, the fraction of heterozygous loci was highest in the outcrossing genotype of *E. paniculata *from Brazil. The selfing genotypes from Jamaica and Nicaragua appear to retain some residual heterozygosity, despite their predominantly autogamous mating systems. We also found evidence of read mapping errors in 8469 loci where more than one selfer appeared heterozygous. Possible explanations for these read-mapping errors include, sequencing errors, alternative splicing and most likely paralogy. With our current data and the available methods we have no way of determining their relative contribution to read mapping errors.

Sequencing of multiple paralogous transcripts will generate short reads that are similar or identical to many other reads derived from different loci. As a result, when there has not been sufficient divergence between gene copies, reads may be erroneously mapped to the reference sequence. One possible explanation for false heterozygosity is that repetitive elements, for example, conserved motifs in gene families, may be difficult or impossible to assemble into long contigs using current technology. As a result, a fraction of the original short reads may not have been assembled by Oases, or included in our consensus transcriptome. However, if they share enough similarity with a paralog in the transcriptome they may be incorrectly mapped. This could explain, in part, why the original Oases assemblies contain so many short contigs (< 100 bp, see Figure 1). If there is divergence between paralogous loci, incorrectly mapped reads may create a signature similar to heterozygosity. For future analyses it is critical that potentially paralogous sequences are identified because evolutionary inferences from non-orthologous genes are misleading. Although our approach does not allow us to unambiguously characterize all paralogous sequences it has provided a useful method for detecting single copy transcripts.

Using a conservative set of 5011 transcripts for which there was no evidence of paralogy, based on homozygosity in all three selfers and presence in all four genotypes, we determined the number of SNPs between each pair of genotypes. As expected, *E. paniculata *samples were more differentiated from *E. paradoxa *than with one another (Table 4). The patterns of divergence among *E. paniculata *genotypes reflect relationships previously reported (see figure three [18]). Specifically, the Nicaraguan selfing genotype is more similar to the outcrossing Brazilian genotype than it is to the selfing Jamaican genotype, despite more similar biogeographical origins and mating systems. This suggests that the Nicaraguan population is more closely related, or more recently derived from the Brazilian population. Further, when we calculate nucleotide polymorphism across 5011 sequences for the three *E. paniculata *genotypes the value we obtained (*θ_W _*= 0.0104) is comparable to our previously published species-wide estimate of total diversity (*θ_W _*= 0.0101 [40]), based on 10 nuclear-derived EST loci assayed in samples of 225 individuals from 26 populations. This evidence supports the validity of our SNP detection method.

### Expression & enrichment

There was a weak trend indicating that the selfing genotypes of *E. paniculata *were more correlated in gene expression; however, this difference was not significant using bootstrapping to generate 95% confidence intervals. All three genotypes of *E. paniculata *retain highly correlated gene expression despite phenotypic divergence and geographic isolation. The slight elevation in correlated expression between the two selfing genotypes of *E. paniculata *may be caused by a small number of genes that are differentially expressed in both selfing genotypes (see below), although overall patterns of expression during flower development appear to remain largely conserved. This may be because we combined all stages of flower development in our assays and a more careful dissection of expression in each stage individually could potentially reveal different patterns of gene expression in selfers and outcrossers.

Enrichment tests of our annotated transcriptomes demonstrated that genes that were differentially expressed in selfers exhibit a paucity of particular gene classes. This can be interpreted as the conservation of expression of these gene functions, which are rarely differentially expressed. Of the 313 GO categories found to be significantly enriched among all differentially expressed genes only 21 were found to be over-abundant, and 20 of these 21 categories were over-represented in genes absent or expressed at lower levels in the two selfers. Therefore, it appears that many of the differences common to the selfing lineages of *E. paniculata *are associated with reductions of gene expression in floral tissue. This may be related to the convergence of floral traits in these two lineages, both of which have much smaller, less pigmented flowers, with reduced pollen production compared to the outcrossing genotype. 96 of the 108 gene classes that are expressed at a low level in the selfers are under-represented but 5 of the 12 over-represented GO categories were associated with the regulation of cell development and structure. Selfing flowers display modified stamen positions and floral instability including twisted, fused or missing perianth parts, whereas outcrossing plants rarely display these floral modifications [42]. It is possible that the changes in gene expression we have documented influence the regulation of cell growth and division and are responsible for changes to floral morphology that characterize selfing populations. If so, these regulatory loci could be used as a set of candidate genes to investigate aspects of the evolution of the selfing syndrome.

### Differentially expressed floral genes in selfers

By sampling different stages of floral development up to and including anthesis we were able to sequence and annotate a large number of florally expressed genes. In total 812 genes with the GO annotation 'reproduction' were identified, which is a large fraction of the number reported for *Arabidopsis thaliana *in which 1184 genes for reproduction have been documented [43]. The lower number of genes in our annotation is not unexpected because we did not include tissue from reproductive stages after flowering, such as fruit and seed development. Within the GO category 'reproduction' we found 269 genes involved in floral development, similar to the number that has been annotated in *A. thaliana *(323 genes). Of particular significance are the floral development genes that are differentially expressed in the three selfing genotypes (Table 5), several of which affect structures that are modified in the selfers. Anther development and filament elongation are influenced by *AFB2 *[44], *ARF6, ARF8 *[45], *BAM1 *[46], *GID1c*, [47]*myb24 *[48], *PGP1 *[44] and *PTR1 *[49] genes in *A. thaliana*, and pollen maturation and pollen tube growth are altered by *AFB2, CSDL3 *[50], *CER1 *[51], *POP2 *[52], *myb24 *[48]and *TIR1 *[44]. *ERL1 *plays an important roles in normal anther lobe formation and anther cell differentiation [53] and mutants in the *ERECTA *gene family have reduced lateral organ size and abnormal flower development, including defects in petal polar expansion and carpel elongation [54]. We also identified a number of genes involved in flowering time including *DCL1 *[55], *PGI1 *[56], *SHK1 *[57], *SUF4 *[58]. Lastly, all of the differentially expressed genes that influence ovule development were significantly up-regulated, including *ARF6, ARF8 *[45], *OVA9 *[59], *PEP *[60]. Significantly, most of the candidate genes discussed above cause alterations to attractive structures (perianth) and male function (stamen development) consistent with the relaxation of selection for showy flowers, reduced allocation to pollen production and the loss of herkogamy (stigma-anther separation) through filament elongation of stamens. In contrast, the requirement for functional ovules to maintain seed fertility in selfers may explain the apparent absence of changes to gene expression in female traits.

## Conclusions

We have shown that short-read sequencing can be used to characterize the transcriptomes of multiple individuals for use in comparative studies. We were able to assemble as many contigs as other sequencing methods by *de novo *assembly, but our contigs were on average substantially longer. By comparing sequences from related individuals we generated a consensus transcriptome. This allowed us to make SNP genotype calls and provided a method for detecting paralogous sequences. Discerning among copies of paralogous sequences presents a major challenge to the current technology and requires either technological or analytical solutions to discern among different members of gene families or duplicates. However, despite these complications we annotated > 80% of contigs and identified thousands of putative orthologs, many of which are differentially expressed. We identified 22 genes that were differentially expressed in selfers and which have developmental functions that suggest a role in the evolution of the selfing syndrome. This sequencing effort has generated a valuable resource of coding DNA for a non-model species. The transcriptome sequences will help in future studies of changes in the genetic architecture involved in the transition from outcrossing to selfing and also for identifying the genes controlling heterostyly.

## Authors' contributions

The study was conceived by RWN and SCHB. The molecular lab work was carried out by RWN. Sequence assembly and annotation and expression analyses were carried out by RWN. MS contributed programming expertise and to methods for implementing genotyping. All authors read and approved the final manuscript.
